# Development of a murine tumor-infiltrating lymphocyte therapy model for cholangiocarcinoma

**DOI:** 10.1093/jimmun/vkaf242

**Published:** 2025-09-16

**Authors:** Megen C Wittling, Frances J Bennett, Emilie A K Warren, Kailey M Oppat, Megan M Wyatt, Jacklyn N Hammons, Yuan Liu, Shishir K Maithel, Chrystal M Paulos, Gregory B Lesinski

**Affiliations:** Division of Surgical Oncology, Department of Surgery, Winship Cancer Institute, Emory University, Atlanta, GA, United States; Department of Microbiology and Immunology, Emory University, Atlanta, GA, United States; Division of Surgical Oncology, Department of Surgery, Winship Cancer Institute, Emory University, Atlanta, GA, United States; Division of Surgical Oncology, Department of Surgery, Winship Cancer Institute, Emory University, Atlanta, GA, United States; Division of Surgical Oncology, Department of Surgery, Winship Cancer Institute, Emory University, Atlanta, GA, United States; Division of Surgical Oncology, Department of Surgery, Winship Cancer Institute, Emory University, Atlanta, GA, United States; Department of Microbiology and Immunology, Emory University, Atlanta, GA, United States; Department of Hematology and Medical Oncology, Winship Cancer Institute, Emory University, Atlanta, GA, United States; Biostatistics Shared Resource, Winship Cancer Institute of Emory University, Atlanta, GA, United States; Division of Surgical Oncology, Department of Surgery, Winship Cancer Institute, Emory University, Atlanta, GA, United States; Division of Surgical Oncology, Department of Surgery, Winship Cancer Institute, Emory University, Atlanta, GA, United States; Department of Microbiology and Immunology, Emory University, Atlanta, GA, United States; Department of Hematology and Medical Oncology, Winship Cancer Institute, Emory University, Atlanta, GA, United States

**Keywords:** cholangiocarcinoma, Tumor Infiltrating Lymphocytes (TILs), Adoptive Cell Transfer (ACT), orthotopic mouse model, PD-L1 blockade

## Abstract

Tumor-infiltrating lymphocyte (TIL) therapy is a promising approach, earning U.S. Food and Drug Administration approval in patients with anti–PD-1–resistant melanoma. Extending TIL therapy to patients with cholangiocarcinoma (CCA), an aggressive and largely immune-refractory cancer, is an emerging area of interest. However, cost and manufacturing complexity constrain clinical scalability of TIL therapy for CCA, underscoring the need for a murine model to optimize efficacy. Here, we established a novel orthotopic model of TIL therapy for CCA and tested a new ex vivo expansion strategy. We first characterized the immune landscape of orthotopic CCA and then compared 2 TIL expansion methods: (1) a conventional protocol using CD3 agonist stimulation (CD3 TILs) and (2) a tumor antigen–based protocol using irradiated autologous CCA cells to enrich for tumor-reactive TILs (Tumor Ag TILs). Tumor Ag TILs displayed superior tumor lysis in vitro compared to CD3 TILs. While both TIL products engrafted in vivo, Tumor Ag TILs showed enhanced persistence. Despite this, monotherapy with either TIL product alone had only a modest impact on tumor growth rate, and infused cells had upregulation of inhibitory checkpoint receptors, including PD-1. Further investigations demonstrated that the in vivo antitumor efficacy of both Tumor Ag TILs and CD3 TILs was enhanced when combined with PD-L1 inhibitor therapy. Altogether, our study establishes a preclinical platform for modeling CCA TIL therapy, identifies a rational combination strategy that potentiates TIL efficacy, and provides the field with a foundation to advance adoptive T-cell transfer development for CCA and related solid tumors.

## Graphical Abstract

**Figure:**
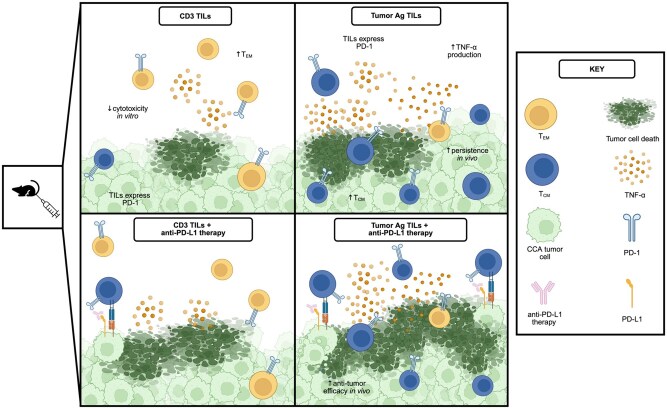

## Introduction

Cholangiocarcinoma (CCA) is an aggressive malignancy that arises in the biliary tract of patients, with a dismal 5-year overall survival rate of less than 20%.^1^ CCA can be either intrahepatic (iCCA) or extrahepatic and is often detected at later stages, with less than 40% of patients presenting with resectable tumors at the time of diagnosis.^2^^,^^3^ A 6-month course of adjuvant chemotherapy is recommended for patients with resectable tumors.^3^^,^^4^ However, for patients with unresectable disease, first-line therapy consists of systemic chemotherapy (gemcitabine and cisplatin) combined with PD-1/PD-L1 blockade therapy, based on results of the recent phase 3 TOPAZ-1 and KEYNOTE-966 trials.^3^^,^^5^^,^^6^ Incorporation of immunotherapy into front-line treatment for CCA patients marks an incremental but important advance, with improved overall survival at 2 years compared to chemotherapy alone.^6^ Despite advances, primary and secondary resistance to immunotherapy remains a major issue and limits efficacy in patients with CCA.

There is a concerted effort to enhance the efficacy of immunotherapy in CCA patients through combination approaches. The goal to overcome immunotherapy resistance in patients with CCA has led to numerous multicenter clinical trials exploring combination strategies, including trials that combine immune checkpoint blockade therapy with MAPK-targeted inhibition or approaches that concurrently administer multiple immunotherapy modalities.^7–12^ However, such combinations have yet to demonstrate improvement in oncologic survival outcomes.^7^^,^^8^^,^^13^ Several redundant mechanisms likely contribute to the limited efficacy of immunotherapy in patients with CCA. These include suppressive cytokines and cells within the tumor, and for iCCA tumors, their presence in the liver is associated with immune tolerance and heterogenous genomic features that link with distinct tumor microenvironments (TMEs).^14–16^ Clearly, identifying more effective immunotherapy approaches remains an unmet need for patients with CCA.

Adoptive T cell transfer (ACT) therapy is a promising strategy to enhance immune-mediated recognition and elimination of CCA. ACT encompasses therapies that harness the antitumor activity of T cells—either by engineering them with a T cell receptor (TCR) or chimeric antigen receptor, or by expanding naturally arising tumor-infiltrating lymphocytes (TILs). Two ACT products were U.S. Food and Drug Administration–approved for solid tumors: lifileucel (a TIL-based therapy to treat patients with metastatic melanoma) and afamitresgene autoleucel (a TCR therapy for metastatic synovial sarcoma).^17^^,^^18^ TIL therapies for new indications are on the horizon. In CCA, there are provocative reports illustrating the promise of TIL therapy. For example, in one patient with progressive, metastatic CCA, TILs targeting a mutated tumor antigen (ERBB2IP) were identified, expanded, and administered as TIL therapy—resulting in complete tumor regression ongoing for more than a decade.^19^ In another case report, a patient with CCA achieved a partial response with ipilimumab followed by TIL therapy and subsequent nivolumab.^20^ Given the excitement caused by these findings, a phase 2 study is currently recruiting patients to evaluate the efficacy of TIL therapy for biliary tract cancers (NCT03801083). These findings support ACT as a viable therapeutic option for patients with CCA and underscore the need for its continued refinement.

Challenges remain for adapting exogenous administration of T cells to patients with solid tumors, including TIL persistence, trafficking, antigen selection, and off-target effects. These barriers all enforce the need to generate experimental models that allow for more rigorous efforts to optimize CCA TIL therapies.^21^ While murine TIL models for sarcoma, colon adenocarcinoma, and melanoma have contributed to new ways to manufacture more advanced ACT products, there are no murine TIL therapy models for cholangiocarcinoma.^22^^,^^23^ Herein, we generated the first CCA TIL mouse model that can be used as a platform to innovate and refine ACT therapy in the preclinical setting. We outline effective strategies to overcoming the hurdles of expanding antigen-specific T cells from TILs in a relevant CCA murine model and assess the cooperative effects of combining ACT with checkpoint blockade therapy. Last, this work advances efforts to adapt and refine TIL therapies for CCA, with implications for future treatment options for patients.

## Materials and methods

### Orthotopic injection of cholangiocarcinoma

The University of Rochester CCA (URCCA) 4.3 cell line (gift from Dr David Linehan) is a syngeneic murine tumor cell line derived from spontaneously occurring *LSL-Kras^G12D^*; *Trp53^Flox/Flox^*; *Alb-Cre* (KPPC) mouse iCCA.^24^ URCCA4.3 was cultured in RPMI 1640 with 1% antibiotic:antimycotic (Gemini Bioproduct, #400-101), 1% L-glutamine, and 10% FBS in flasks coated with collagen 0.05 mg/mL (Collagen I Rat Tail, Corning, #354236). Prior to injection into mice, URCCA4.3 cells were confirmed negative for mycoplasma. A total of 200,000 URCCA4.3 cells were mixed with extracellular matrix and/or Matrigel at a 1:1 ratio. C57BL/6 immunocompetent mice were then placed under general anesthesia using 3% isoflurane. Extended-release buprenorphine (1 mg/kg) was administered subcutaneously for analgesia. The mouse abdomen was prepped and draped in the sterile fashion, and adequate liver exposure was achieved via an open midline incision. The URCCA4.3 cell mixture was orthotopically injected directly into the superficial liver capsule, injecting a total of 12 µL intrahepatically. Abdominal incisions were closed with absorbable suture and staples. Staples were removed by postoperative day 10. Tumors were then grown for approximately 12 days prior to harvesting for infiltrative immune cell isolation and characterization and to initiate TIL expansion.

### Mice

Female C57BL/6 mice were purchased from the Jackson Laboratory (strain #000664) and co-housed as 5 mice per cage. Female B6.PL-Thy1a/CyJ mice were purchased from the Jackson Laboratory (strain #000406) and co-housed as 5 mice per cage. Female Rag1^−/−^ mice (B6.129S7-Rag1tm1Mom/J) were purchased from the Jackson Laboratory (strain #034159) and co-housed as 5 mice per cage. All animal work was approved by the Institutional Animal Care and Use Committee at Emory University with additional support from Emory’s Division of Laboratory and Animal Resources.

### Orthotopic tumor collection and processing

As described above, C57BL/6 mice bearing orthotopic URCCA4.3 tumors were euthanized approximately 12 days after undergoing surgery, at which time tumors were harvested and subsequently processed. Tumors were minced and digested using collagenase (Roche, #11088866001), dispase (Stem Cell Technologies, #07923), and liberase enzymes (Sigma-Aldrich, #5401119001) prior to undergoing further processing using the GentleMACS Tissue Dissociator and incubation at 37 °C for 30 minutes. Resultant cell suspensions were then strained over a 70-µm filter (Fisher Scientific, #08-771-2) and then either stained with flow antibodies to characterize the infiltrative immune cells present in the tumor or further processed for initiation of an ex vivo TIL culture.

### TIL culture

Following tumor tissue processing, immune cells were isolated from the resultant digested tumor cell suspension with an EasySep Mouse CD45 Positive Selection Kit (StemCell, #18945). Isolated CD45^+^ immune cells then either underwent further flow characterization or were plated at a density of 2 × 10^6^ cells per well in a 24-well plate. TIL cultures were expanded with high-dose IL-2 (6,000 IU/mL) (Teceleukin, #1035-0490) and via one of 2 separate protocols using either CD3 agonist (CD3 TILs) or irradiated URCCA4.3 tumor cells with or without splenocytes at a 5:1 T-cell to irradiated URCCA4.3 plus splenocyte ratio (Tumor Ag TILs). CD3 TILs received CD3 Ab at 1 μg/mL (Ultra-Leaf purified antimouse CD3ε, BioLegend, #100340). Tumor Ag TILs received URCCA4.3 tumor cells irradiated at 100 Gy together with or without C57BL/6 splenocytes irradiated at 10 Gy. These cells were added at a ratio of 5:1 (T cell:tumor plus splenocyte). TILs were then expanded for approximately 12 days with additional IL-2 (6,000 IU/mL) added when cells were split.

### Flow cytometry

Flow cytometry was performed at multiple stages, including on digested URCCA4.3 tumor cell suspensions before and after undergoing CD45 positive selection sort to characterize immune cells in the TME of orthotopic murine URCCA4.3 tumors. Additionally, flow cytometry was performed on expanded TILs prior to adoptive transfer, as well as blood, tumor-draining lymph nodes, and tumors of mice post–adoptive transfer. For analysis of cytokine production using flow cytometry, samples were incubated for 4 hours with Brefeldin A (5 μg/mL; BioLegend, #420601) and monensin (2 μM; BioLegend, #420701) with or without PMA (50 ng/mL; Sigma, #P1585-1mg) and ionomycin (1 μg/mL; Sigma, #I3909-1mL). After completion of incubation, or immediately if not evaluating cells for cytokines, samples were stained with a viability stain (BioLegend, #423101 and #423107) at a 1:1,000 ratio in PBS for 15 minutes at room temperature. Following the viability stain, extracellular protein staining at a 1:500 ratio was completed in FACS buffer (PBS + 2% FBS) for 15 minutes. The FoxP3/Transcription Factor Kit (Invitrogen, #00-5523-00) was then used to stain for intracellular antibodies, per the provided instructions, with antibodies at a 1:100 ratio. A Cytek Aurora 5 Laser with UV System instrument was used to collect flow cytometry data (Cytek Biosciences). Analysis of flow cytometry data was completed using FlowJo software (BD Biosciences, version 10.10.0).

### Flow cytometry antibodies

Flow cytometry antibodies used to characterize the tumor-infiltrating immune cells, tumor-infiltrating T cells, and expanded TILs prior to and after transfer ex vivo and in various tissues (lymph node, tumor, peripheral blood) included the following: Zombie UV (BioLegend), Zombie Aqua (BioLegend), F4/80 (clone: T45-2342, BD Biosciences), NK1.1 (clone: PK136, BioLegend), CD11c (clone: N418, BioLegend), CD45 (clone: 30-F11, BioLegend), CD4 (clone: GK1.5, BioLegend), CD3 (clone: 17A2, BioLegend), CD19 (clone: 1D3, BD Biosciences), Ly6G (clone: 1A8, BioLegend), Ly6C (clone: HK1.4, BioLegend), CD11b (clone: M1/70, BioLegend), CD8 (clone: 53-6.7, BioLegend), T-bet (clone: 4B10, BioLegend), FoxP3 (clone: MF-14, BioLegend), CD69 (clone: H1.2F3, BioLegend), Lag-3 (clone: C9B7W, BioLegend), PD-1 (clone: 29F.1A12, BioLegend), CD44 (clone: IM7, BioLegend), Tim-3 (clone: B8.2C12, BioLegend), TOX (clone: NAN448B, BD Pharmigen), TCF-1 (clone: C63D9, Cell Signaling), Ki-67 (clone: 11F6, BioLegend), CD62L (clone: MEL-14, BioLegend), CD39 (clone: Duha59, BioLegend), TNF-α (clone: MP6-XT22, BioLegend), Tim-3 (clone: RMT3-23, BioLegend), and CTLA-4 (clone: UC10-4B9, BioLegend).

### Cytotoxicity assays

TIL cytolytic activity on both primary and repeat secondary tumor exposure was evaluated and measured using an in vitro impedance-based assay (xCELLigence, Agilent). First, xCELLigence 96-well plates were coated with collagen (0.05 mg/mL) (Collagen I Rat Tail, Corning #354236). A baseline plate reading was obtained, per manufacturer instructions; then, 5,000 URCCA4.3 tumor cells (target cells) were added to each well in 100 µL of Complete Media.

#### Primary Target Cell Challenge

Approximately 24 hours following addition of target/tumor cells, effector TILs from 2 separate expansion protocols, CD3 TILs and Tumor Ag TILs, were added to respective wells at predetermined effector cell (TIL) to target cell (URCCA4.3) (E:T) ratios of 0.5:1, 1:1, 5:1, and 10:1. A full-lysis control using 1% Triton X-100 was also performed. Following the addition of TIL effector cells, impedance was measured every 15 minutes for a total duration of approximately 50 hours. Changes in impedance were measured and used to calculate the normalized cell index and percent cytolysis using RTCA software (ACEA Biosciences).

#### Secondary Target Cell Rechallenge

To evaluate TIL cytolytic activity on repeat tumor exposure, an additional xCELLigence 96-well plate was prepared with collagen and coated with new URCCA4.3 target cells, as described above. Unlike the primary target cell challenge, TILs of both cohorts that were tested in the primary xCELLigence experiment were then transferred into the wells of the newly plated, treatment-naïve target URCCA4.3 cells at the same E:T ratios (0.5:1, 1:1, 5:1, 10:1). Similarly, a full-lysis control was performed. Impedance measurements were recorded every 15 minutes for approximately 50 hours. Normalized cell index and percentage cytolysis were again calculated using RTCA software.

An additional experiment tested the potential role of splenocyte addition to the TIL cytotoxicity. Primary and secondary challenge experiments as described above were again performed using CD3 TILs and Tumor Ag TILs that had been expanded in the presence or absence of irradiated splenocytes (Fig. S4). TILs were expanded per the respective expansion protocols with or without irradiated splenocytes.

### IL-2 complex preparation

Antihuman IL-2 antibody (R&D Systems, #MAB602) was mixed with recombinant human (rh) IL-2 (Teceleukin, #1035-0490) (per injection: antihuman IL-2 antibody 7.5 mg + rhIL-2, 1.5 mg) in sterile PBS, incubated for 15 minutes at 37 °C, and stored at –80 °C until use.

### In vivo administration of TIL product as monotherapy

Subcutaneous tumors were established in Rag1^−/−^ mice via injections of 1 × 10^6^ URCCA4.3 CCA cells. Tumor area was monitored throughout the experiment via caliper measurement at a minimum of twice per week. Seventeen days following URCCA4.3 subcutaneous injection, all mice had established tumors and received 3 Gy total body irradiation (TBI). Mice were randomized to one of 3 treatment arms: CD3 TILs (*n* = 10), Tumor Ag TILs (*n* = 9), or no treatment (*n* = 11). One day after receiving TBI, mice randomized to treatment arms were administered 5 × 10^6^ TILs (CD3 TILs or Tumor Ag TILs) via tail vein injection. Intraperitoneal IL-2 complex was administered to mice on the day of ACT administration, in addition to day 2 and day 4 post-ACT. Twenty days post-ACT, peripheral blood was collected directly into EDTA-coated tubes (Sarstedt, #16.444.100). Serum was isolated and stored at –80 °C. Blood was further processed using RBC lysis buffer (BioLegend, #420302) for a total duration of 5 minutes at room temperature. Complete Media containing FBS was then added to inactivate further lysis. Cells were stained for flow cytometry, as described above. Twenty-five days post-ACT, mice were euthanized, and tumors, draining inguinal lymph nodes, and peripheral blood were collected. Tumor and blood were processed as described above. Lymph nodes were processed into a single-cell suspension by dissociation over a 70-µm filter. Flow cytometry was then performed.

### In vivo administration of combination therapy with TIL product plus early anti–PD-L1 therapy

Subcutaneous URCCA4.3 CCA tumors were established and monitored in immune competent C57BL/6 mice, as described above. Six days after tumor administration, palpable, established tumors were visually confirmed, and all mice received 5 Gy TBI. Mice were randomized to one of 6 treatment arms (*n* = 3 to 4 mice/group): anti–PD-L1 alone, isotype control alone, CD3 TILs plus anti–PD-L1, CD3 TILs plus isotype control, Tumor Ag TILs plus anti–PD-L1, or Tumor Ag TILs plus isotype control. CD3 TILs and Tumor Ag TILs were processed and expanded from orthotopic tumors in B6 CD45.1 mice (B6.SJL-Ptprca Pepcb/BoyJ) that had grown for approximately 12 days and were then expanded for approximately 12 days, as described above. One day following TBI, mice randomized to TIL treatment arms received 3 × 10^6^ TILs per mouse via tail vein injection. Mice received intraperitoneal IL-2 complex on the day of ACT administration, in addition to day 2 and 4 following ACT. Isotype control 200 µg/mouse (BioXcell clone LTF-2, #BE0090) and anti–PD-L1 200 µg/mouse (BioXcell clone B7-H1, #BE0101) were administered via intraperitoneal injection to the respective groups on days 6, 8, 11, and 13 after ACT administration. Mice were measured at least twice weekly.

### In vivo administration of combination therapy with TIL product plus late anti–PD-L1 therapy

Subcutaneous injections of 1 × 10^6^ URCCA4.3 CCA cells were given to immune competent C57BL/6 mice. Tumor area was monitored throughout the experiment via caliper measurement at a minimum of twice per week. Six days after tumor administration, all mice received 5 Gy TBI. Mice were randomized to one of 3 treatment arms (*n* = 3 to 5 mice/group): anti–PD-L1 alone, CD3 TILs plus anti–PD-L1, or Tumor Ag TILs plus anti–PD-L1. CD3 TILs and Tumor Ag TILs were processed and expanded from orthotopic tumors in B6 CD45.1 mice (B6.SJL-Ptprca Pepcb/BoyJ) that had grown for approximately 12 days and were then expanded for approximately 12 days, as described above. One day following TBI, mice randomized to TIL treatment arms received 5 × 10^6^ TILs per mouse intravenously. Mice received intraperitoneal IL-2 complex on day of ACT administration, in addition to day 2 and 4 following ACT. Anti–PD-L1 (200 µg/mouse) (BioXcell clone B7-H1, #BE0101) was administered to all groups via intraperitoneal injection on days 30, 32, 34, and 36 post-ACT. Mice were measured at least twice weekly.

### Statistical analysis

Univariate analysis of continuous variables used Mann–Whitney *U* test or Student *t*-test. Kaplan–Meier log-rank analysis was performed to determine association between mice treatment arms and overall survival. For comparison of longitudinal tumor growth in vivo between treatment groups, tumor volume data underwent Box–Cox data transformation^25^ to meet the normality assumption of linear modeling. The resulting transformed data were then used to fit a linear mixed-effect model with days, treatment group, and their interaction as predictors. Subsequently, the tumor growth rates among treatment groups were estimated and compared. The random effect was specified at the mouse level, including random intercepts and growth rates. Variance-covariance matrix was selected as the one which generated the smallest Akaike information criterion. For these studies, a model fitness check was done to examine the normality of residual distribution and equal variance by predictors. Statistical analyses were performed using either GraphPad Prism (version 10.3.1) or SAS/STAT (version 9.4) software. The overall significance was set at *P* < 0.05.

## Results

### Activated T cells are present in murine CCA tumors

**Figure 1.**
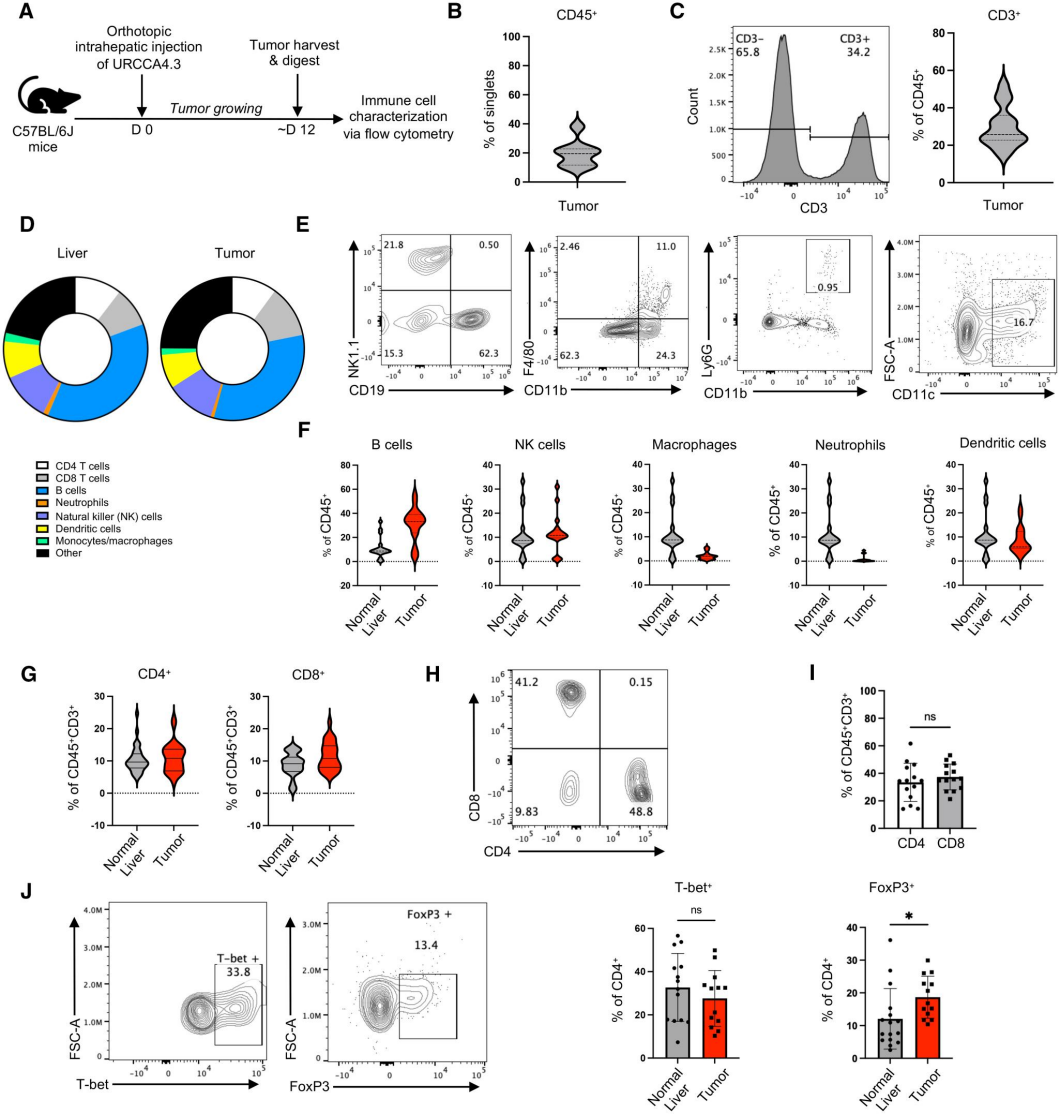
Activated T cells are present in murine orthotopic cholangiocarcinoma models. (A) Orthotopic CCA murine model schematic. CBL57/6J mice were given orthotopic intrahepatic CCA tumors via injection of 2 × 10^5^ URCCA4.3 cells. Tumors were grown for approximately 12 days and then harvested and subsequently CD45 sorted. Flow cytometry was then performed. (B) CD45 expression of single cells. Gated on lymphocytes/single cells (*n* = 8). (C) CD3 expression in the tumor. Gated on lymphocytes/single cells/Live/CD45^+^ (*n* = 14). (D) Immune cells within URCCA4.3 intrahepatic tumors and adjacent liver tissue (average taken of *n* = 14). (E) Immune cell subtypes within the TME. Gated on lymphocytes/single cells/Live/CD45^+^/CD3^−^. (F) B cells (CD3^−^CD19^+^), NK cells (CD3^−^NK1.1^+^), macrophages (CD3^−^CD11b^+^F4/80^+^), neutrophils (CD3^−^CD11b^+^Ly6G^+^), and DCs (CD3^−^CD11c^+^) in orthotopic URCCA4.3 tumors and tumor-adjacent healthy liver (*n* = 17 to 18). (G) CD4 and CD8 expression among CD45^+^CD3^+^ cells in orthoptic URCCA4.3 tumors and tumor-adjacent normal liver (*n* = 7 to 9). (H) Distribution of CD4 and CD8 T cells in URCCA4.3 orthotopic tumors. Gated on lymphocytes/single cells/Live/CD45^+^/CD3^+^/CD4^+^ or CD8^+^. (I) Comparing CD4 and CD8 expression of tumor-infiltrating CD45^+^CD3^+^ cells (*n* = 14). (J) T-bet and FoxP3 expression on CD4 T cells. Representative flow plots from tumor tissue. Gated on lymphocytes/single cells/Live/CD45^+^/CD3^+^/CD4^+^ (*n* = 13 to 16). Experiment independently performed 5 times. Statistical analysis of continuous variables done using Mann–Whitney tests, where statistical significance was defined as *P* ≤ 0.05: ns: not statistically significant (*P* > 0.05), *P ≤ 0.05.

We first defined the immune landscape in aggressive murine orthotopic CCA tumors to assess what immune cells were present that may be expanded as a cellular therapy product. As detailed in Fig. 1A, established orthotopic intrahepatic URCCA4.3 tumors were harvested, and phenotypic features of immune cells within this malignancy were determined via flow cytometry. After tumor digestion and processing, approximately 20% of single cells within the tumor were CD45^+^ (Fig. 1B, Fig. S1A). Immune cells represented about 70% of the live cells present, likely due to mincing of tumors and digestion prior to flow cytometry staining (Fig. S1B, C). Greater than 30% of CD45^+^ cells in the TME expressed the CD3 T-cell marker (Fig. 1C). Additionally, other immune cells were detected, including a large proportion of B cells (∼30%), as well as natural killer cells (NK) (∼10%) and dendritic cells (CD11c^+^) (∼8%) (Fig. 1D). Cells expressing CD11b^+^F4/80^+^ and CD11b^+^Ly6G^+^ were also detected, albeit at a lower frequency (Fig. 1D, E). These populations were found at similar frequency in both the tumor-adjacent liver and tumor itself (Fig. 1D, F). Gating used to detect these populations is displayed in Fig S1D. The proportion of CD4 and CD8 T cells was also similar when comparing tumor-adjacent liver and tumor, and CD4 and CD8 T cells infiltrated the tumors at comparable levels (Fig. 1G–I). Additionally, both Th1 (T-bet^+^) and regulatory T cells (Treg) (FoxP3^+^) were present in the liver and CCA tumor, with about 30% of CD4 T cells expressing transcription factors indicative of Th1 phenotype and approximately 10% displaying a regulatory profile (Fig. 1J). There was an increase in Tregs within the tumor compared to liver, possibly indicative of a more immunosuppressive TME.

**Figure 2.**
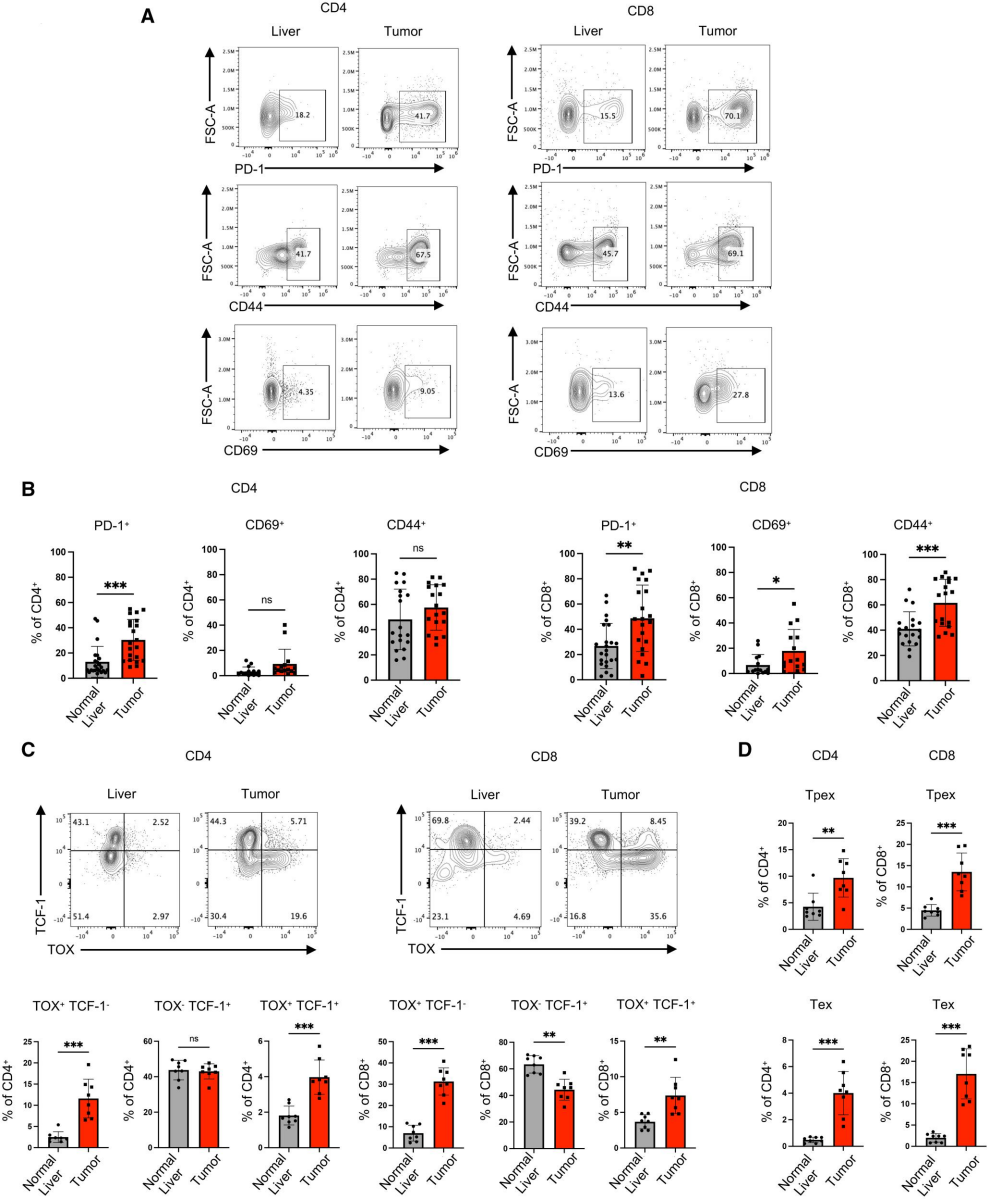
Comparing CD4 and CD8 T cells in healthy, tumor-adjacent liver vs orthotopic intrahepatic URCCA4.3 cholangiocarcinoma TMEs. (A) Representative flow plots comparing PD-1, CD44, and CD69 expression of CD4 and CD8 T cells present in tumor-adjacent liver and tumor. Gated on lymphocytes/single cells/Live/CD45^+^/CD3^+^/CD4^+^ or CD8^+^. (B) Increased activation of T cells in intrahepatic URCCA4.3 tumors (PD-1^+^, CD44^+^, CD69^+^) (*n* = 21 to 23 mice/group). (C) TCF-1 and TOX expression on CD4 and CD8 T cells within tumor-adjacent liver tissue and tumor. Gated on lymphocytes/single cells/Live/CD45^+^/CD4^+^ or CD8^+^ T cells. (*n* = 8 mice/group). Representative flow plots with corresponding bar charts below. (D) Quantification of CD4 and CD8 progenitor exhausted T cells (Tpex) (Tim-3^−^/PD-1^+^/TCF-1^+^/TOX^low/int^) and terminally exhausted T cells (Tex) (Tim-3^+^/PD-1^+^/TCF-1^−^/TOX^+^) (*n* = 8 mice/group) in tumor-adjacent liver and tumor. Experiments in (A) and (B) were performed 5 times. Experiments corresponding to (C) and (D) were performed twice. Statistical analysis of continuous variables used Student *t*-test or Mann–Whitney test, where statistical significance was defined as *P* ≤ 0.05: ns: not statistically significant (*P* > 0.05), *P ≤ 0.05, **P ≤ 0.01, ***P ≤ 0.001.

Interestingly, T cells infiltrating the tumor harbored a more activated profile distinct from T cells in adjacent liver (Fig. 2A, B). For example, PD-1 was expressed at significantly higher levels on both CD4 and CD8 T cells in the tumor compared with adjacent non-tumor-involved liver (Fig. 2B). Tumor-infiltrating CD8 T cells also expressed more CD44 and CD69 than adjacent liver (Fig. 2B). To further assess the memory profile of T cells in the tumor compared to normal tissue (tumor-adjacent liver), we compared expression of TOX, a transcription factor critical for maintenance of exhausted T cells,^26^ and TCF-1, a transcription factor associated with T cell self-renewal and stemness.^27^ CD4 and CD8 T cells expressing TOX (TOX^+^TCF-1^+^ and TOX^+^TCF-1^−^) were significantly elevated in the tumor relative to tumor-adjacent liver (Fig. 2C), implying they may be more exhausted within the TME. To further assess these exhaustion profiles, we determined the levels of progenitor exhausted (Tpex) (Tim-3^−^/PD-1^+^/TCF-1^+^/TOX^low/int^) and exhausted (Tex) T cells (Tim-3^+^/PD-1^+^/TCF-1^−^/TOX^+^) (see gating strategy in Fig. S2). CD4 and CD8 Tpex and Tex cells were present at higher levels within the tumor compared to tumor-adjacent liver (Fig. 2D).

### Expansion and characterization of murine CCA–derived TILs

**Figure 3.**
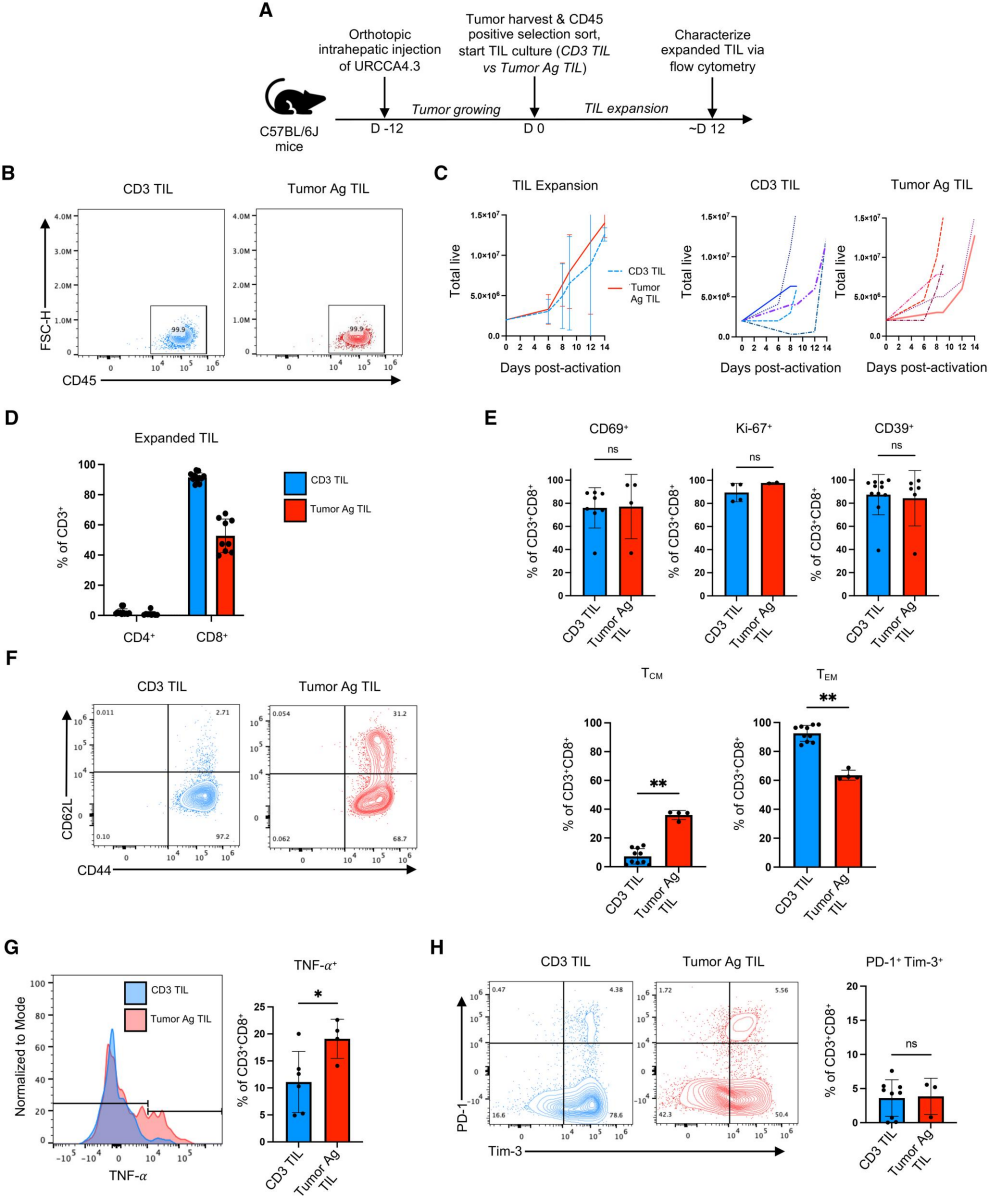
T cells can be expanded from murine cholangiocarcinoma tumors. (A) Schematic of TIL expansion protocol. Intrahepatic orthotopic URCCA4.3 tumors were harvested and digested, and CD45^+^ cells were isolated via positive selection sort. T cells were then expanded in the presence of high-dose IL-2 and either CD3 agonist (CD3 TILs) or irradiated tumor and splenocytes (Tumor Ag TIL). After ∼12 days of expansion, expanded TILs were characterized via flow cytometry. (B) Expanded cells were CD45^+^. Gated on lymphocytes/single cells/Live. (C) Growth curve of TIL product expansion stratified by expansion protocol (CD3 TIL, Tumor Ag TIL) (*n* = 5). (D) CD8 T cells preferentially expand. Gated on lymphocytes/single cells/Live/CD45^+^. (E) Expanded TILs were activated and proliferating regardless of expansion method (CD69^+^, Ki-67^+^, CD39^+^). (F) Tumor Ag TIL expansion promotes CD8 central memory T-cell phenotype (CD44^+^CD62L^+^). (G) Tumor Ag TILs produced greater levels of TNF-α compared to CD3 TIL. (H) Regardless of expansion method, expanded TILs were not terminally differentiated based on low levels of PD-1 and Tim-3 dual positivity. TIL expansion experiments were performed 5 times. Statistical analysis of continuous variables used Mann–Whitney test, where statistical significance was defined as *P* ≤ 0.05: ns: not statistically significant (*P* > 0.05), *P ≤ 0.05, **P ≤ 0.01.

Murine models represent a platform to optimize TIL manufacturing for subsequent ACT therapy. For example, TILs harvested from mice with various malignancies have been successfully expanded ex vivo^22^^,^^28–30^ and enabled researchers to test ideas about how to improve ACT therapies with enhanced memory or metabolic properties. However, to date, there are no reports evaluating the feasibility of expanding TILs from murine CCA models. Because T cells with activated phenotypes were present, we hypothesized that TILs isolated from this model could be expanded for future ACT therapy. To test this idea, CCA tumors were processed and intratumoral immune cells isolated and plated with 6,000 IU/mL of IL-2 and either expanded in the presence of a CD3 agonist (CD3 TILs) or irradiated tumor with or without splenocytes (Tumor Ag TILs). TILs were expanded for approximately 12 days and then profiled using flow cytometry (Fig. 3A).

Most expanded cells expressed CD45 (>99%) (Fig. 3B), and both protocols (CD3, Tumor Ag) yielded successful expansion of TIL products (Fig. 3C). CD8 T cells preferentially expanded over CD4 T cells in both groups (Fig. 3D). Regardless of expansion protocol, CD8 TILs were in an activated state, marked by heightened CD69 and CD39 expression, and were proliferating (Ki-67^+^) (Fig. 3E). When examining the expanded CD8 TILs, the Tumor Ag TIL protocol supported the propagation of T cells with an increased central memory phenotype (T_CM_) (CD44^+^CD62L^+^) compared to the CD3 TIL expansion (median T_CM_: Tumor Ag TILs: 37.40% vs CD3 TILs: 6.40%; *P* = 0.002). Both methods yielded a prominent effector CD8 T cell population (CD44^+^CD62L^−^) (Fig. 3F). Additionally, Tumor Ag TILs secreted more TNF-α compared to CD3 TILs (*P* = 0.04) (Fig. 3G). Last, regardless of expansion method, few expanded TILs were terminally differentiated (Tim-3^+^PD-1^+^) (*P* = 0.60) (Fig. 3H).

### TILs expanded in the presence of tumor-specific antigens exhibit superior cytotoxic effects in vitro

**Figure 4.**
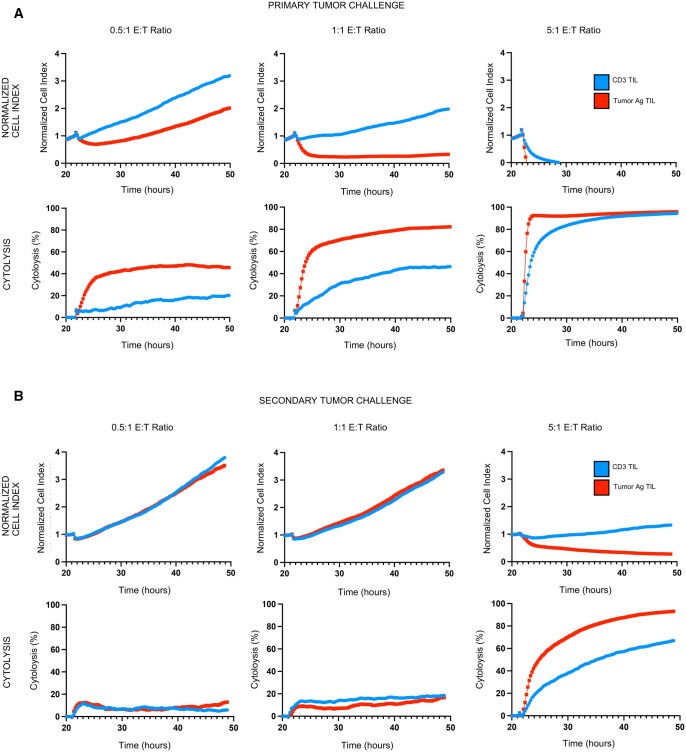
Tumor Ag TILs demonstrated enhanced cytolytic activity against URCCA4.3 using an in vitro impedance-based assay. (A) Primary tumor challenge: CD3 TILs or Tumor Ag TILs were added to an in vitro impedance-based cytolytic assay (xCELLigence) with URCCA4.3 cells at various E:T ratios and monitored for changes in normalized cell index and cytolysis. Target cells (URCCA4.3) were plated and then effector cells (TIL) were added at approximately the 24-hour time point. Cytolysis and changes to the normalized cell index were measured over time. (B) Secondary tumor challenge: Treatment-naive URCCA4.3 cells were then plated in unused xCELLigence plates, and TILs from the primary tumor challenge were added at various E:T ratios. Cytolysis and changes to normalized cell index were measured over time. Primary and secondary tumor challenge of CD3 TIL and Tumor Ag TIL xCELLigence experiments were reproduced 3 times.

Potent cytotoxicity and antitumor effects of T-cell products are dependent on antigen specificity and persistence.^31–33^ We posited that murine TILs expanded in the presence of tumor-specific antigen (Tumor Ag TILs) would exhibit superior cytotoxicity and mediate robust antitumor effects compared to those expanded via more traditional expansion methods (CD3 TILs). To test this concept in vitro, TILs isolated from murine CCA orthotopic intrahepatic tumors were expanded for approximately 12 days using the CD3 or Tumor Ag TIL protocols described. Antitumor activity of the expanded TILs from each cohort was then evaluated for cytotoxic activity using an impedance-based cytotoxicity assay (xCELLigence). Fig. 4A represents the changes in normalized cell index and corresponding measures of cytolysis observed by CD3 or Tumor Ag TILs against URCCA4.3 during the initial primary tumor challenge at varying E:T ratios (0.5:1, 1:1, 5:1). At higher E:T ratios of 5:1 and 10:1, both CD3 and Tumor Ag TILs demonstrate similarly efficacious levels of tumor killing, nearing 100% cytotoxicity (Fig. 4A, Fig. S3). Interestingly, however, Tumor Ag TILs demonstrated superior killing at the lower E:T ratios (0.5:1 and 1:1) compared to the CD3 TILs (Fig. 4A), illustrating enhanced cytolytic activity when TIL expansion occurred in the presence of irradiated URCCA4.3 cells as an antigen source. Finally, both TIL products were tested for ability to sustain and remount an antitumor response after chronic exposure to tumor. To do this, we reexposed the same TILs from the first tumor challenge to newly plated, treatment-naïve tumor cells (Fig. 4B). We hypothesized that Tumor Ag TILs would prevail, marked by lasting cytotoxicity compared to CD3 TILs upon secondary tumor rechallenge. Although slightly dampened compared to the primary tumor challenge, both TIL products lysed tumor again on secondary tumor challenge. At the lower E:T ratios of 0.5:1 and 1:1, neither group elicited a strong antitumor response, but when assessed at the 5:1 and 10:1 E:T ratios, Tumor Ag TILs mounted superior cytolysis (Fig. 4B, Fig. S3B).

Additionally, we evaluated the role of the antigen-presenting cells (APCs) (irradiated splenocytes) in the Tumor Ag TIL cohort to confirm that this was not the cause for the superior cytolytic activity observed. We hypothesized that the enhanced tumor killing observed by Tumor Ag TILs was due to TIL expansion in the presence of tumor-specific antigens from irradiated URCCA4.3 tumor, rather than the presence of the irradiated APCs. To test this idea, we isolated immune cells from orthotopic URCCA4.3 tumors, as described above, and expanded both CD3 and Tumor Ag TILs in the presence or absence of irradiated splenocytes for a total of 4 different expansion groups (Fig. S4). TIL cytotoxicity was then tested in vitro using xCELLigence primary and secondary URCCA4.3 tumor challenges, like above. Our findings confirmed the superior cytotoxicity by Tumor Ag TILs, and more importantly, demonstrate that even in the presence or absence of APCs during TIL expansion, Tumor Ag TILs still perform superiorly to CD3 TILs (Fig. S4). These findings reinforce the conclusion that it is specifically TIL expansion in the presence of tumor-specific antigens that propagates their potent tumor-killing properties.

### TILs expanded in presence of tumor antigens persist longer in mice

**Figure 5.**
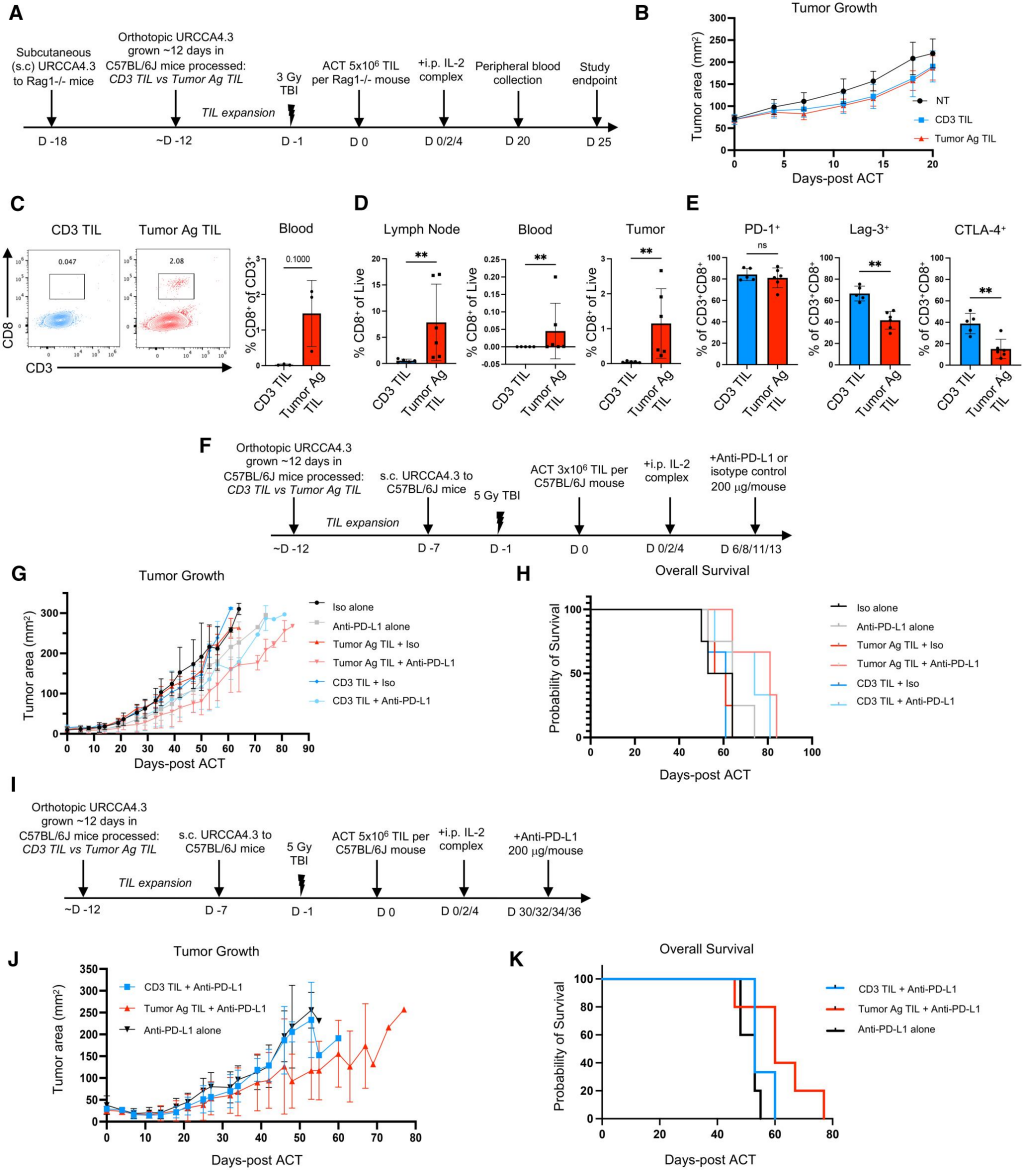
Cholangiocarcinoma TILs delay tumor growth and improve antitumor immunity when combined with anti–PD-L1 therapy. (A) Adoptive T-cell transfer therapy (ACT) schematic corresponding to (A–E). Survival and tumor size were monitored to endpoint, day 25 following TIL receipt, at which time flow analysis was performed on the tumor-draining lymph node, peripheral blood, and tumor. (B) Tumor area (mm^2^) from day of ACT administration to day 20 post-ACT (CD3 TIL mice, *n* = 10; Tumor Ag TIL mice, *n* = 9, no treatment [NT], *n* = 11). (C) Peripheral blood TIL engraftment on day 20 post-ACT. Gated on lymphocytes/single cells/Live/CD45^+^/CD3^+^CD8^+^. (D) Persistence of CD8^+^ TILs in the lymph node, peripheral blood, and tumor 25 days post-ACT. (E) PD-1, Lag-3, and CTLA-4 expression on CD8 TILs in the peripheral blood 25 days post-ACT. (F) Combination ACT plus early anti–PD-L1 therapy schematic, corresponding to (F–H). (G) Tumor area (mm^2^) from day of ACT to day 84 post-ACT (*n* = 3 to 4 mice/group). (H) Overall survival stratified by treatment arm in mice with subcutaneous URCCA4.3. (I) Combination ACT plus late anti–PD-L1 therapy schematic, corresponding to (I–K). (J) Tumor area (mm^2^) from day of ACT administration to day 77 following ACT, stratified by treatment arm (*n* = 3 to 5 mice/group). (K) Overall survival stratified by treatment arm in mice with subcutaneous URCCA4.3. Each respective in vivo experiment performed once. Statistical analysis of continuous variables performed using Mann–Whitney test. Statistical significance defined as *P* ≤ 0.05: ns: not statistically significant (*P* > 0.05), **P ≤ 0.01. Kaplan–Meier log-rank analysis was performed to determine association between treatment arm and overall survival and tumor growth rates analyzed as described in the Materials and methods.

After confirming cytolytic activity in vitro, we tested the efficacy of the respective TIL products in vivo. We hypothesized that TIL products, regardless of expansion method, would demonstrate antitumor activity. However, based on our in vitro results, we posited that TILs expanded in the presence of tumor-specific antigens (Tumor Ag TILs) would be more effective than CD3 TILs. We first assessed the efficacy of TIL products without the influence of endogenous host immune cells in a simple in vivo system using immunodeficient recipient mice. For these studies, Rag1^−/−^ mice bearing URCCA4.3 tumors were infused with either CD3 TILs or Tumor Ag TILs and then treated with cytokine support using IL-2 complex (Fig. 5A). As a negative control, additional mice were not treated. Tumor progression was transiently slowed in mice given Tumor Ag TIL therapy (*P* = 0.048) and those receiving CD3 TILs (*P* = 0.113) when compared to controls. However, no significant difference in treatment response was observed between mice that received Tumor Ag versus CD3 TIL products (*P* = 0.65; Fig. 5B). Additional investigation revealed a trend toward better engraftment of Tumor Ag TILs versus CD3 TILs in the blood (median CD8^+^ % of CD3^+^ TILs in peripheral blood 20 days post-ACT: CD3 TILs: 0.02% versus Tumor Ag TILs: 1.92%; *P* = 0.10) (Fig. 5C). Moreover, persistence of Tumor Ag TILs was significantly greater in mice 25 days post-ACT in the tumor, blood, and tumor-draining lymph nodes of mice compared to conventional CD3 TILs (Fig. 5D). Inhibitory markers, particularly PD-1, were expressed on both TIL products and persisted in the peripheral blood 25 days post-ACT (Fig. 5E). While both TIL products expressed PD-1, CD3 TILs displayed higher levels of additional inhibitory receptors, including Lag-3 and CTLA-4 (Fig. 5E). These results suggest that Tumor Ag TILs are less exhausted and may be more responsive to checkpoint blockade than CD3 TILs, providing a strong rationale to explore combination therapies targeting the PD-1/PD-L1 axis concurrent with TIL therapy in more traditional immunocompetent model systems.

### TILs plus anti–PD-L1 therapy improve immunity to CCA

Encouraged by a recent phase 2 clinical trial demonstrating durable responses to TIL therapy combined with pembrolizumab in patients with advanced gastrointestinal malignancies,^34^ we investigated whether a similar strategy could be adapted in our preclinical CCA model. Our rationale stemmed from the elevated PD-1 on persistent Tumor Ag TILs following ACT, implying they remained antigen experienced, yet poised for reinvigoration, after their comparatively lower expression of inhibitory markers Lag-3 and CTLA-4 relative to CD3 TILs. We hypothesized that Tumor Ag TILs would be further improved with anti–PD-L1 therapy, in turn enhancing overall survival compared to either monotherapy or CD3 TILs plus anti–PD-L1 therapy. To evaluate this question, immunocompetent, syngeneic C57BL/6 mice bearing URCCA4.3 tumors were lymphodepleted with 5 Gy TBI 6 days post–tumor inoculation. One day later, mice were infused with either CD3 or Tumor Ag TILs grown from B6 CD45.1 mice, followed by treatment with IL-2 complex and anti–PD-L1 (or isotype control antibody) (Fig. 5F).

Using protocols described above, CD3 or Tumor Ag TILs were derived from orthotopic CCA tumors. Treatment with anti–PD-L1, either alone (*P* = 0.0053) or in combination with either of the TIL therapies (*P* = 0.0009), significantly delayed tumor progression (Fig. 5G). Tumor Ag TIL therapy combined with PD-L1 blockade significantly improved survival compared to Tumor Ag TILs administered with isotype control antibody (*P* = 0.048) (Fig. 5H). Additionally, when either TIL product was combined with the PD-L1 therapy, the dual treatment led to an increased delay in tumor growth compared with PD-L1 alone (*P* = 0.0436) or with TIL alone (*P* = 0.0053), further demonstrating the potential improved impact between these 2 treatments (Fig. 5G). There was also a trend toward improved survival in the Tumor Ag TIL + PD-L1 group compared to the no treatment + isotype group (*P* = 0.068). These findings suggest a favorable interaction between Tumor Ag TILs and PD-L1 blockade, potentially driven by preserved TIL functionality and enhanced reactivation following checkpoint inhibition.

Given that PD-1 expression remained elevated on both TIL products 25 days post-ACT, we next tested whether delayed administration of anti–PD-L1 therapy could further enhance outcomes. Mice bearing established tumors were preconditioned with 5 Gy TBI and infused with either CD3 TILs or Tumor Ag TILs. Twenty-eight days later, anti–PD-L1 therapy was initiated (Fig. 5I). Tumor Ag TILs combined with delayed checkpoint blockade again trended toward the greatest delay in tumor progression (*P* = 0.066; Fig. 5J). Median survival in this group reached 60 days, compared to 53 days for CD3 TILs plus anti–PD-L1 or anti–PD-L1 alone, although this trend did not reach statistical significance (*P* = 0.12; Fig. 5K). Still, the modest survival benefit, delayed tumor growth, and enhanced Tumor Ag TIL engraftment suggest that these TILs remain responsive to checkpoint inhibition and could benefit from temporally optimized combination regimens. Altogether, these data highlight the utility of expanding TILs in the presence of tumor antigen and provide a rationale for integrating delayed PD-1/PD-L1 blockade to bolster ACT efficacy in CCA.

## Discussion

This report highlights the feasibility of isolating and expanding TILs from clinically relevant, orthotopic murine CCA tumors. This TIL therapy model will be a valuable foundation for the scientific community to further innovate in the ACT therapy space. Herein, we demonstrate successful expansion of TILs with potent and sustained in vitro cytotoxic killing, in addition to efficacy in vivo, particularly when administered in combination with immunotherapy targeting the PD-1/PD-L1 pathway. We first identified the proportion of T cells present in these tumors to assess their inherent potential for expansion in the context of what other immune subsets were present in orthotopic CCA tumors. Importantly, a substantial proportion of both CD4 and CD8 T cells were present in the tumors—highlighting the feasibility of their expansion using this model. Further, we validated that these tumor-infiltrating T cells were more activated than T cells in adjacent liver tissue, based on PD-1, CD44, and CD69 expression. We also detected increased progenitor exhausted as well as exhausted CD4 and CD8 T cells in the tumors compared to adjacent healthy liver, indicating a pool of both less-exhausted progenitor cells, as well as an exhausted subset that may have cytotoxic qualities. These activated T cells could be expanded ex vivo and could lyse tumor upon primary and secondary tumor exposure in vitro. Additionally, Tumor Ag TILs possessed a central memory phenotype and a trend toward improved antitumor properties compared to the more traditionally expanded CD3 TILs. Last, the rate of tumor growth was inhibited in mice given Tumor Ag TILs with anti–PD-L1 therapy, resulting in improved overall survival in aggressive CCA models.

Two distinct expansion protocols were tested after processing and isolating CD45^+^ cells from orthotopic CCA tumors. First, the CD3 TIL protocol applied more traditional methods using a CD3 agonist for activation and high-dose IL-2 for expansion,^35^^,^^36^ akin to that used in clinical settings. However, identifying efficient TIL expansion methods that produce tumor-reactive TILs remains a clinical challenge. Specifically, neoantigen-reactive TILs have shown promise in patients with biliary tract cancer and other malignancies.^19^^,^^34^^,^^37^ In metastatic melanoma, response to TIL therapy correlates with the presence of more tumor-reactive T cells,^35^ in addition to receipt of a tumor-specific TIL product with stem-like features.^38^ Additionally, recent reports of a phase 2 clinical trial evaluating the role of ACT therapy against various gastrointestinal malignancies found that approximately 24% of patients treated with a neoantigen-specific TIL product combined with pembrolizumab experienced an objective response.^34^ Given this promising response to a neoantigen-specific TIL therapy, we sought to develop a TIL expansion protocol that would preferentially expand tumor-reactive TILs, hypothesizing that this product would adopt greater cytolytic properties, which we named Tumor Ag TILs. Tumor Ag TILs were expanded with IL-2 and tumor antigens in the form of irradiated CCA. This novel methodology was developed from studies showing that tumor lysate can be combined with TILs for CCA clinically in one case report,^39^ and in which tumor-specific cells T cells targeting sarcoma were generated in part by addition of 100 Gy irradiated tumor and splenocytes added to T cells in culture.^40^ Also, in patients, addition of APCs loaded with mutated p53 or RAS peptides selectively expanded tumor-reactive TILs in a novel expansion method termed NeoExpand,^41^ again underscoring the potential benefit of tumor antigen addition during TIL generation.

Interestingly, regardless of expansion method, both yielded similar quantities of TIL product. CD8 T cells preferentially expand, and effector CD8 T cells were prevalent in both types of expanded TILs. Additionally, we showed that the expanded TIL products were activated (CD69^+^, CD39^+^) and proliferating (Ki-67^+^) but not terminally differentiated based on limited coexpression of Tim-3 and PD-1. Interestingly, the Tumor Ag TIL expansion method promoted expansion of CD8 T cells with a T_CM_ phenotype and enhanced secretion of TNF-α as compared to the more traditional CD3 TIL expansion.

When comparing cytotoxic activity of TIL products in vitro, the Tumor Ag protocol generated a more potent and cytotoxic TIL product with superior cytotoxic activity at lower E:T ratios on both primary tumor exposure and secondary tumor challenge. Such enhanced tumor killing is likely attributable, at least in part, to greater levels of TNF-α produced, and the larger, more prevalent central memory CD8 population in Tumor Ag TILs. This is a subset of CD8 T cells that has previously demonstrated superior tumor killing compared to effector CD8 T cells.^42^^,^^43^ Additionally, prior studies in other solid tumors demonstrate that TIL products generated from antigen-specific T cells promote a more potent antitumor response.^31^^,^^44^ These results further support our hypothesis that the greater efficacy and antitumor response on primary and secondary tumor exposure in vitro is attributable to expansion of TILs that are educated with tumor antigen during expansion and possess central memory–type features. Finally, cytolytic activity was preserved and remained superior when Tumor Ag TILs were expanded without APCs (irradiated splenocyte addition), supporting that Tumor Ag TIL cytotoxicity is likely driven by both increased antigen-specificity and the qualities previously mentioned.

We evaluated the cytotoxicity of these TIL therapies in vivo, finding that Tumor Ag TILs persisted better long-term following ACT administration in both the lymph node, peripheral blood, and tumor compared to CD3 TILs. Although our findings show only a moderate delay in tumor progression when mice were treated with TIL therapy alone, we identified several checkpoint inhibitors expressed on our TIL products. Thus, we improved therapeutic efficacy by targeting the PD-1/PD-L1 pathway, in part due to the high PD-1 expression seen on the transferred TILs. Additionally, applying anti–PD-L1 therapy offers clinical relevance given not only its role as standard therapy for patients with unresectable, metastatic CCA, but also in its use in clinical trials combined with TILs for gastrointestinal malignancies.^6^^,^^34^ In our in vivo model, combining Tumor Ag TILs with anti–PD-L1 therapy consistently reduced tumor growth and trended toward improved overall survival, regardless of the timing of checkpoint blockade. Notably, Tumor Ag TILs with anti–PD-L1 outperformed anti–PD-L1 monotherapy, whether checkpoint inhibition was administered early or delayed relative to ACT, highlighting the potential potency of these TIL products. Recent clinical trials for CCA utilized immunotherapy targeting PD-1/PD-L1, and a recent phase 2 clinical trial combined neoantigen-specific TILs with pembrolizumab in patients with various gastrointestinal malignancies.^5^^,^^6^^,^^34^ Therefore, further exploration of combination therapies such as this, first in the preclinical setting, and then translating to the bedside, is warranted. Ongoing work in our laboratory aims to enhance TIL therapy through alternative polarization strategies and combinations with TME-modulatory agents, among other approaches. This report is therefore timely, as our TIL model offers a clinically relevant system where TIL therapy shows efficacy but also clear potential for further optimization.

Cholangiocarcinoma harbors an immunosuppressive, desmoplastic tumor microenvironment in patients, consisting of large amounts of myeloid cells, fibroblasts, and other cell types that dampen antitumor T-cell responses.^45^ The tumor line used in this study originated from a spontaneously arising intrahepatic CCA (KPPC) that harbored abundant myeloid cells.^24^^,^^46^ Our analysis of the TME of this KPPC-derivative cell line, URCCA4.3, demonstrated fewer infiltrating myeloid cells when tumors were examined following orthotopic intrahepatic injection. We acknowledge this as a possible limitation in these proof-of-principle studies. We reason this could be explained by either the distinct time point that was used for TIL isolation, or the more rapid growth rate of these tumors. Nonetheless, we posit that our model system remains influential in its ability to allow both isolation and examination of tumor-infiltrating T cells. Importantly, this model provides a foundation for advancing and optimizing TIL therapies for CCA, with the long-term goal of translating these therapies to benefit patients. With successful TIL therapy generation, we are now armed with the ability to study innovative ways to improve these TIL products. Future studies will explore novel ways to combine immune checkpoint blockade with our TIL product, as well as to generate or select more functional and resilient T cells.

## Conclusions

In conclusion, this report represents the first successful isolation and expansion of TILs from an orthotopic murine cholangiocarcinoma model. These data and this new CCA TIL model will enable further study and optimization of cellular therapy in relevant preclinical models. This model thus supports a long-term goal of developing clinical-grade cell therapy products for testing in patients with CCA. Our findings demonstrate that TIL expansion in the presence of tumor antigen generates a more cytotoxic, reactive TIL therapy in vitro. Additionally, treatment with TILs combined with targeting the PD-1/PD-L1 pathway delayed tumor progression compared to PD-L1 alone, and Tumor Ag TILs with delayed PD-L1 therapy had the most improved overall survival. Further research will focus on how to efficiently manufacture and continually refine TIL products in the presence of CCA antigen for optimal antitumor activity, while also exploring possible synergistic combination therapies that utilize TILs and immunotherapy.

## Supplementary Material

vkaf242_Supplementary_Data

## Data Availability

Data are included in the main and supplementary figures. Additional information and raw data are available from the corresponding authors upon reasonable request.
