# Supplementary material for: Development of a murine tumor-infiltrating lymphocyte therapy model for cholangiocarcinoma
Source: J Immunol. 2025 Sep 16;215(1):vkaf242. doi: 10.1093/jimmun/vkaf242 (PMC12704411; doi:10.1093/jimmun/vkaf242)
Supplement: vkaf242_Supplementary_Data [file vkaf242_supplementary_data.zip › SupplementalFigure-3.pdf]

Supplemental Figure 3:

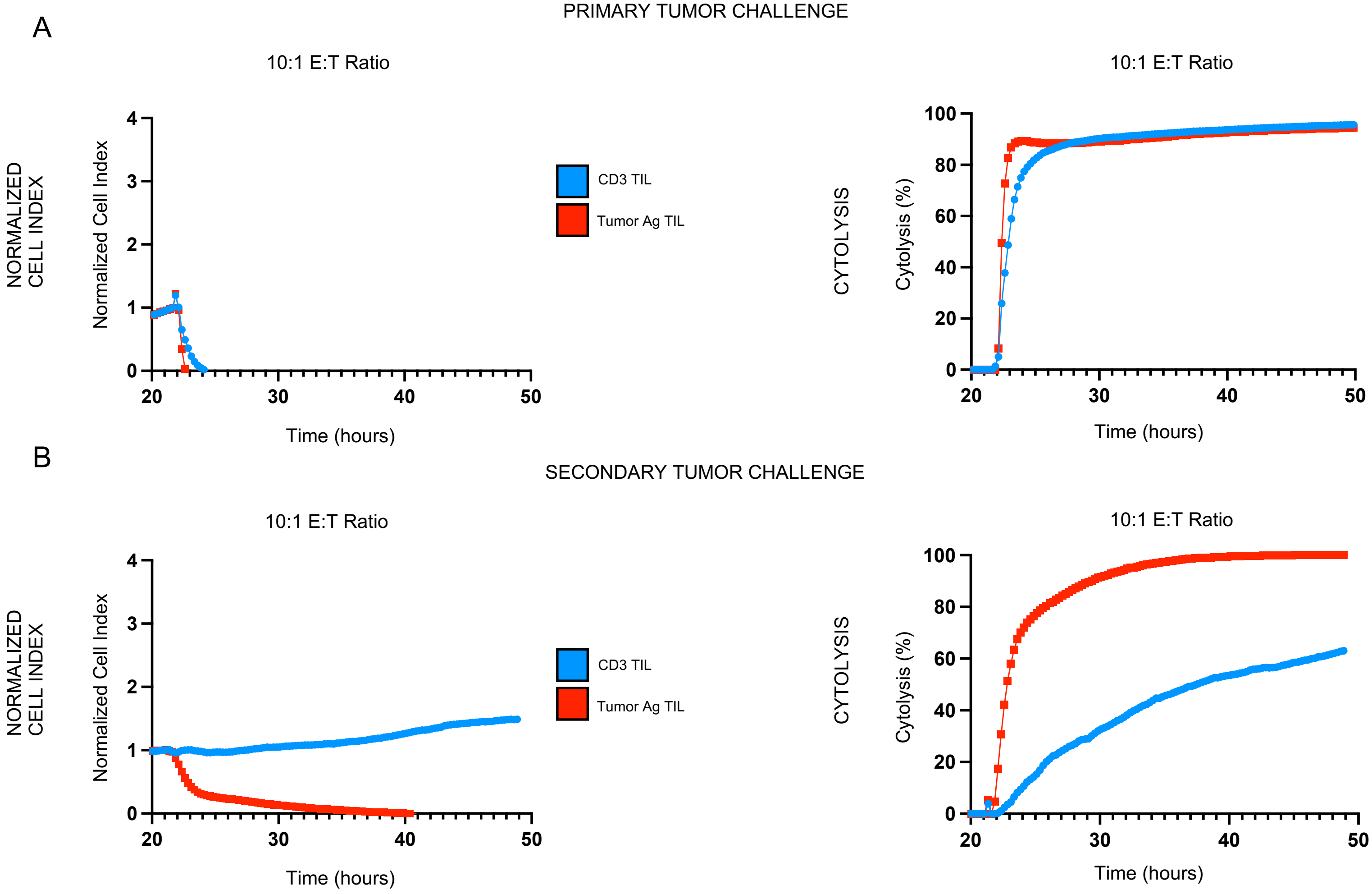

Supplemental Figure 3: ***Tumor Ag TIL demonstrated enhanced cytolytic activity against URCCA4.3 using an impedance-based assay at high effector to target cell ratios.*** **(A)** Primary Tumor Challenge: CD3 TIL or Tumor Ag TIL were added to an impedance-based cytolytic assay (xCELLigence) with URCCA4.3 CCA cells at a 10:1 effector cell to target cell (E:T) ratio and monitored for changes in normalized cell index and level of cytolysis over time. Effector cells (TIL) were added approximately 24-hours following URCCA4.3 cell plating. **(B)** Secondary Tumor Challenge: TIL of each expansion group that were exposed to URCCA4.3 during primary tumor challenge were re-exposed to freshly-plated URCCA4.3 CCA cells. Changes in normalized cell index and level of cytolysis over time were evaluated.
