# Supplementary material for: Development of a murine tumor-infiltrating lymphocyte therapy model for cholangiocarcinoma
Source: J Immunol. 2025 Sep 16;215(1):vkaf242. doi: 10.1093/jimmun/vkaf242 (PMC12704411; doi:10.1093/jimmun/vkaf242)
Supplement: vkaf242_Supplementary_Data [file vkaf242_supplementary_data.zip › SupplementalFigure-1.pdf]

Supplemental Figure 1:

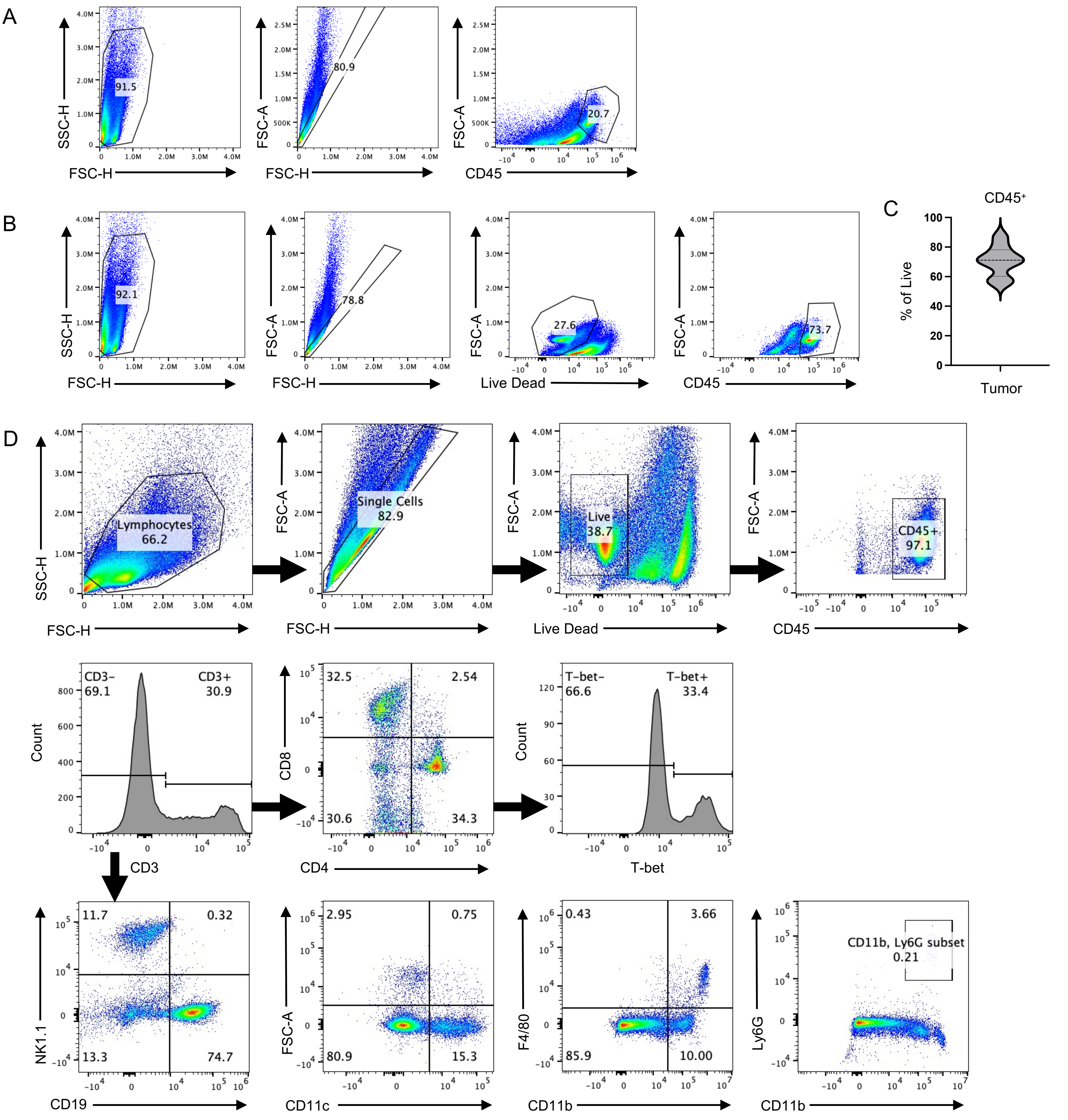

Supplemental Figure 1: ***Gating Strategy to Assess Immune Cell Tumor Infiltration Via Flow Cytometry.*** Orthotopic intrahepatic URCCA4.3 cholangiocarcinoma tumors from CBL57/6J mice were harvested after growing for ~12 days, digested, and then CD45+ immune cells within the tumor microenvironment were characterized by flow cytometry. **(A)** Gating strategy for analysis of CD45 expression of single cells. All cells gated on lymphocytes/single cells. **(B)** Gating strategy for CD45 expression of live cells. All cells gated on lymphocytes/single cells/Live. **(C)** CD45 expression of live cells within the tumor (n=8). **(D)** Gating strategy for characterizing the infiltrating immune cell subtypes in tumor and liver tissue. All cells gated on lymphocytes/single cells/Live/CD45$^+$. Cells were then gated on CD3. CD3$^+$ cells were then gated on CD4 and CD8 for further characterization of CD3$^+$CD4$^+$ or CD3$^+$CD8$^+$ T cells, respectively. Th1 CD4$^+$ T cells were identified based on T-bet positivity and regulatory T cells based on Fox-P3 positivity. CD3$^-$ cells were then further analyzed to characterize B cells (CD19$^+$NK1.1$^-$), natural killer cells (CD19$^-$NK1.1$^+$), dendritic cells (CD11c$^+$), macrophages (CD11b$^+$F4/80$^+$), and neutrophils (CD11b$^+$Ly6G$^+$).
