# Supplementary material for: Development of a murine tumor-infiltrating lymphocyte therapy model for cholangiocarcinoma
Source: J Immunol. 2025 Sep 16;215(1):vkaf242. doi: 10.1093/jimmun/vkaf242 (PMC12704411; doi:10.1093/jimmun/vkaf242)
Supplement: vkaf242_Supplementary_Data [file vkaf242_supplementary_data.zip › SupplementalFigure-2.pdf]

Supplemental Figure 2:

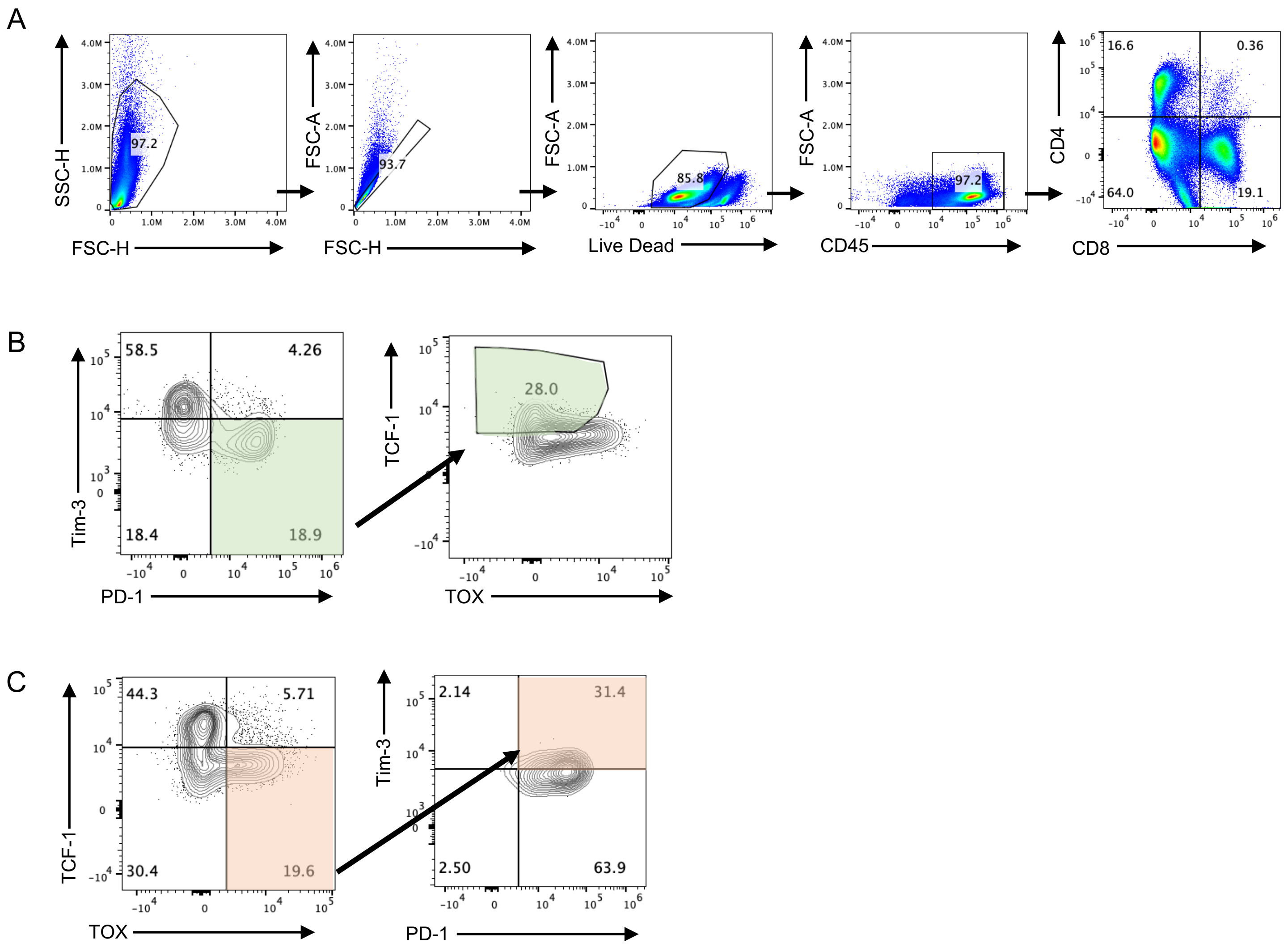

Supplemental Figure 2: ***Gating Strategy for Progenitor Exhausted and Terminally Exhausted T cells.*** **(A)** Gating schema for selection of CD4 and CD8 T cells. **(B)** Gating strategy for progenitor exhausted CD4 and CD8 T cells (Tpex) (Tim-3^{-}/PD-1^{+}/TCF-1^{+}/TOX$^{low/int}$). **(C)** Gating strategy for terminally exhausted CD4 and CD8 T cells (Tex) (Tim-3^{+}/PD-1^{+}/TCF-1^{-}/TOX^{+}).
