# Supplementary material for: Development of a murine tumor-infiltrating lymphocyte therapy model for cholangiocarcinoma
Source: J Immunol. 2025 Sep 16;215(1):vkaf242. doi: 10.1093/jimmun/vkaf242 (PMC12704411; doi:10.1093/jimmun/vkaf242)
Supplement: vkaf242_Supplementary_Data [file vkaf242_supplementary_data.zip › SupplementalFigure-4.pdf]

Supplemental Figure 4:

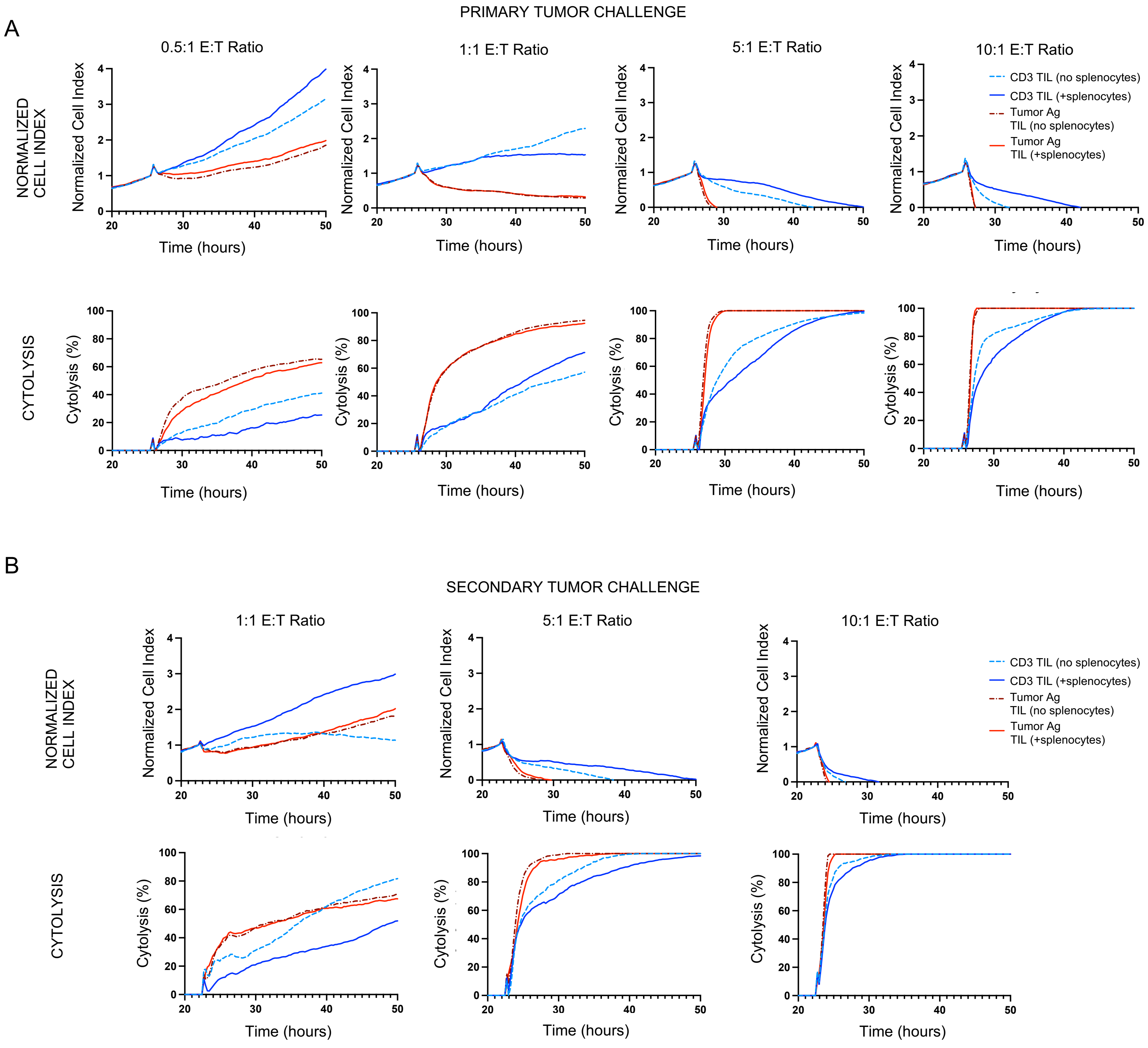

Supplemental Figure 4: ***Addition of splenocytes to TIL during expansion does not impact cytolytic activity against URCCA4.3, as measured by an impedance-based assay.*** **(A)** Primary Tumor Challenge: CD3 TIL or Tumor Ag TIL were expanded with or without splenocytes and added to an impedance-based cytolytic assay (xCELLigence) with URCCA4.3 CCA cells at various effector cell to target cell (E:T) ratios. Normalized cell index and level of cytolysis were monitored over time. Effector cells (TIL) were added approximately 24-hours following URCCA4.3 cell plating. **(B)** Secondary Tumor Challenge: TIL of each expansion group that were exposed to URCCA4.3 during primary tumor challenge were re-exposed to treatment-naive URCCA4.3 CCA cells. Using the impedance-based assay, changes in normalized cell index and level of cytolysis were evaluated over time. Primary and secondary tumor challenge using xCELLigence with CD3 TIL and Tumor Ag TIL +/- splenocytes during TIL expansion performed once with three technical replicates per condition.
