# Supplementary figures and images for: Development of a murine tumor-infiltrating lymphocyte therapy model for cholangiocarcinoma
Source: J Immunol. 2025 Sep 16;215(1):vkaf242. doi: 10.1093/jimmun/vkaf242 (PMC12704411; doi:10.1093/jimmun/vkaf242)

Visual Abstract

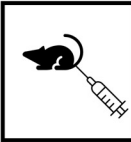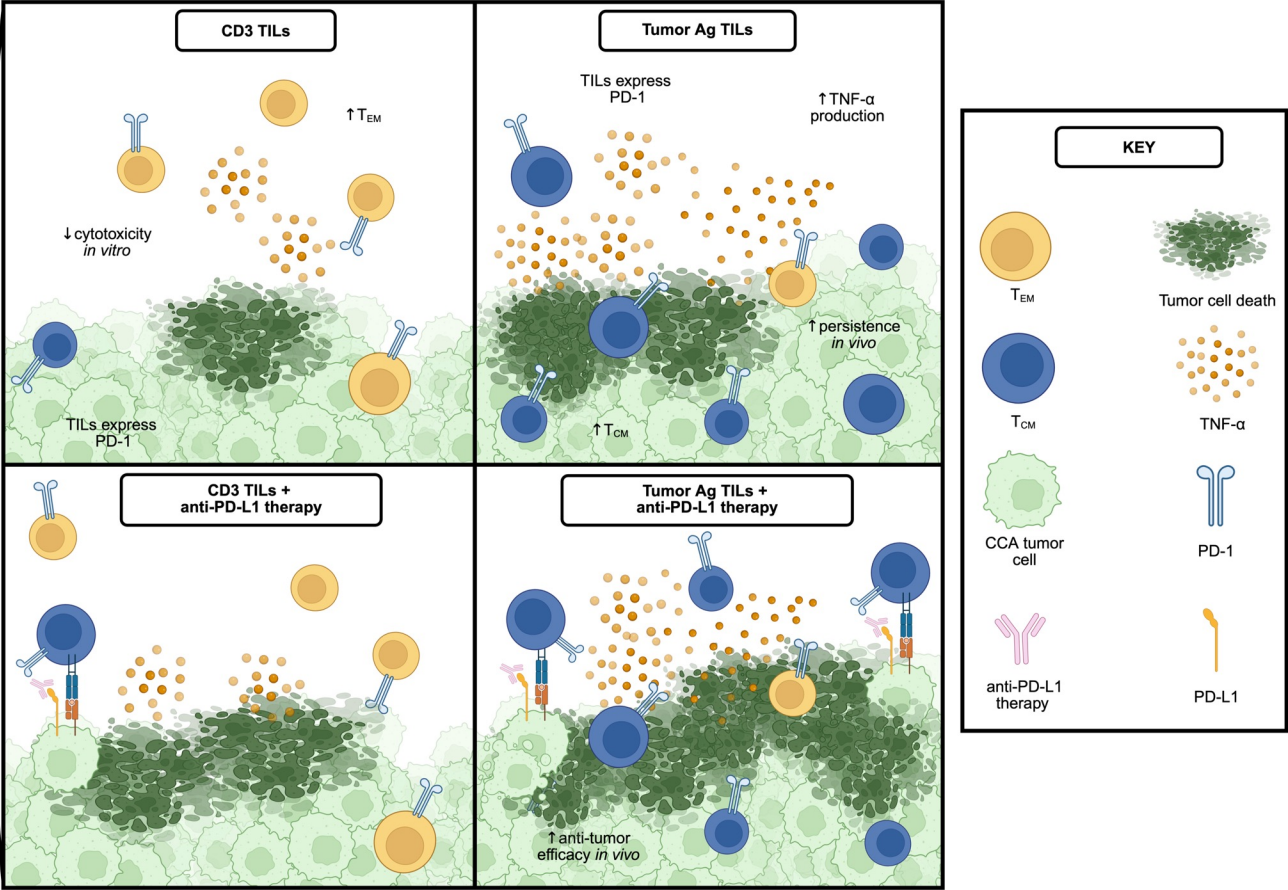

Supplement: vkaf242_Supplementary_Data [file vkaf242_supplementary_data.zip › Visual-Abstract.pdf]
